# Spotlight on Early Career Researchers: an interview with Blake Ushijima

**DOI:** 10.1038/s42003-018-0145-0

**Published:** 2018-10-31

## Abstract

Blake Ushijima is a post-doctoral researcher at Oregon State University, where he studies the microbes causing disease in corals. In this next instalment of our Q&A series, he discusses how he got into marine microbiology and the anxieties of researchers studying coral ecosystems.

**Figure:**
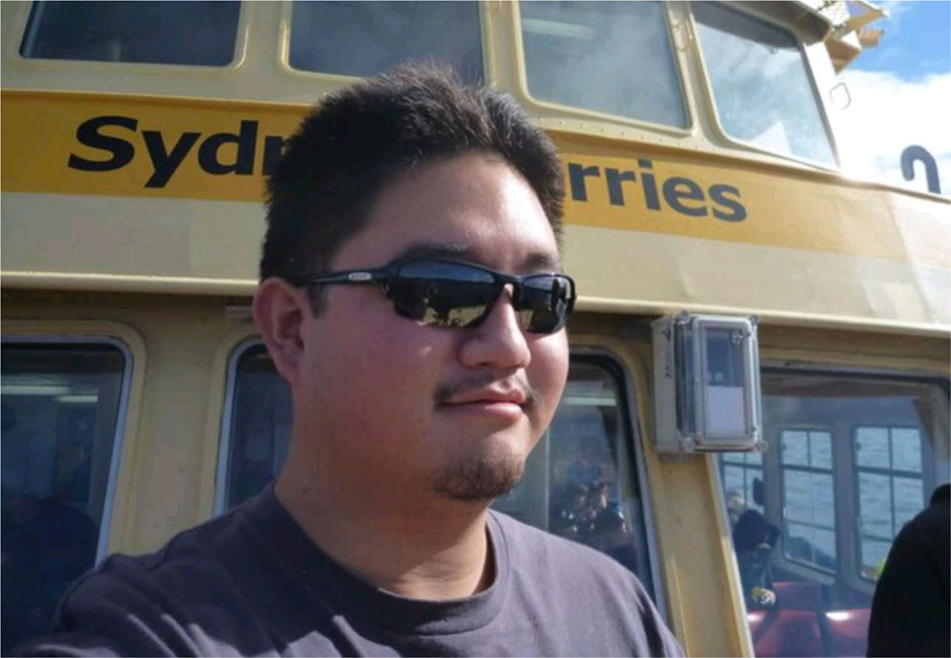
Image credit: Blake Ushijima

Please tell us about your research interests**.**

I am mainly interested in studying bacterial-host and bacterial-bacterial interactions on marine invertebrates. Specifically, I study the pathogenic bacterium *Vibrio coralliilyticus*, which is responsible for mass mortalities of reef-building coral species, and how this pathogen is able kill their hosts and spread in the environment. Additionally, I am interested in the various species of marine bacteria that can colonize coral that can protect their hosts from *V. coralliilyticus* through the production of antibacterial compounds, competition for resources, or other means. By understanding how *V. coralliilyticus* can infect coral, spread throughout the environment, and defend against beneficial bacteria, I hope to develop effective mitigation efforts that could have a positive effect on coral reefs.

What has your journey been to this point?

I’ve always had an interest in marine biology. Growing up in Hawaii and caring for a 200-gallon saltwater aquarium with my grandfather fed my fascination with the invertebrates I could find on the reefs and tidepools. However, it was during this time I was also introduced to the fragility of these marine ecosystems when commercial development sent massive amounts of muddy runoff into the ocean, killing off the reefs along the beach I used to frequent. Then during my undergraduate studies, I became fascinated with microorganisms during the microbiology courses I attended. So, when I came across the opportunity to volunteer as an intern studying the bacteria associated with a common coral species in Hawaii I jumped at the opportunity that melded two of my personal interests. Eventually, this led me into graduate school at the University of Hawaii studying diseases that affected the local coral reefs for my dissertation work. Several major personal losses during my Ph.D. work dampened my workflow for a period, but I was able to pull through and complete my degree while in the process identifying multiple bacterial pathogens responsible for various coral diseases in Hawaii. After graduating, I was hired as a postdoctoral researcher at Oregon State University investigating the pathogens associated with larval and adult Pacific oysters as well as probiotic microorganisms that protect these invertebrates against infection. During my time at OSU, I became involved with a collaboration with the Smithsonian Marine Station in Florida working on the ongoing coral disease outbreak occurring there. Recently, I was awarded a fellowship by the Smithsonian Institute and will be continuing as a postdoctoral fellow there investigating the use of probiotic bacteria to treat diseased coral or to protect healthy specimens from infection.

What are your predictions for your field in the near future?

The recent mass mortalities of coral reefs around the world caused by global climate change and disease outbreaks currently threaten the host organisms that are the focus of my research. The rapidly declining health of multiple major reef systems around the world is and will continue to put an increasing feeling of urgency on the work that I and other coral-focused researchers are conducting. Unfortunately, this current state the of the world leaves me with an overwhelming feeling of uncertainty for my predictions on my field of research. However, I refuse to give up on coral reefs and hope more of the public and world governments recognize the importance of these marine ecosystems.

Can you speak of any challenges that you have overcome?

One challenge is being in a somewhat in a grey area of research that encompasses coral biology and molecular pathogenesis. I don’t focus my research on human pathogens and I am not a coral ecologist, but I study the molecular mechanisms of virulence for coral pathogens and must maintain and study coral in a laboratory. Other early-career researchers in my field and related fields studying non-human pathogens have also expressed similar sentiments of almost being outside of the “norm” for their field. This has resulted in somewhat challenging justifications for granting agencies that fund projects focused on coral ecology or aquaculture research. This challenge has directed me to expand my research topics into diseases of economically important shellfish or human pathogens associated with marine invertebrates, which has been a productive diversification of my research. Additionally, it has been an interesting experience finding and training students that are comfortable with all aspects of coral disease research; I feel it results in a more well-rounded training experience for them. Consequently, this challenge has also given me the opportunity to work with a range of individuals with a diverse set of scientific backgrounds from pathogenesis, ecology, chemistry, and aquaculture.

What advice would you give to your younger self?

You will fail to achieve at 100% of the opportunities you never pursue. Stop underestimating your self-worth, intelligence, and potential. Go after that fellowship, research award, or job posting you thought might be a little too much of a longshot.

What would you say is the best microbe?

Of course, I would have to say *Vibrio coralliilyticus*. The strategies this bacterium has evolved to live in the environment, infecting different hosts, and dealing with microbial competitors has astounded me over the years I’ve studied it.

*This interview was conducted by Senior Editor Dominique Morneau*

